# The role of chronotype and reward processing in understanding social hierarchies in adolescence

**DOI:** 10.1002/brb3.2090

**Published:** 2021-03-01

**Authors:** Judith Lunn, Thomas Wilcockson, Tim Donovan, Frank Dondelinger, Guillermo Perez Algorta, Padraic Monaghan

**Affiliations:** ^1^ Lancaster Medical School Lancaster University Lancaster UK; ^2^ School of Sport, Exercise, and Health Sciences Loughborough University Loughborough UK; ^3^ Centre for Medical Imaging University of Cumbria Carlisle UK; ^4^ Division of Health Research Faculty of Health and Medicine Lancaster University Lancaster UK; ^5^ Psychology Department Lancaster University Lancaster UK

**Keywords:** adolescent social status, chronotype, Reward processing

## Abstract

**Introduction:**

Circadian rhythms shift toward an evening preference during adolescence, a developmental period marked by greater focus on the social domain and salience of social hierarchies. The circadian system influences maturation of cognitive architecture responsible for motivation and reward, and observation of responses to reward cues has provided insights into neurocognitive processes that underpin adolescent social development. The objective was to investigate whether circadian phase of entrainment (chronotype) predicted both reward‐related response inhibition and social status, and to explore whether mediator and moderator relationships existed between chronotype, reward processing, and social status outcomes.

**Methods:**

Participants were 75 adolescents aged 13–14 years old (41 females) who completed an eye tracking paradigm that involved an inhibitory control task (antisaccade task) within a nonsocial reward (Card Guessing Game) and a social reward (Cyberball Game) context. Chronotype was calculated from weekend midsleep and grouped into early, intermediate, and later terciles. Participants indicated subjective social status compared with peers in seven domains.

**Results:**

An intermediate and later chronotype predicted improved inhibitory control in the social versus nonsocial reward context. Chronotype also predicted higher perceived social status in two domains (powerful, troublemaker). Intermediate chronotypes reported higher “Powerful” status whereas later chronotypes were higher on “Troublemaker.” Improved social reward‐related performance predicted only the higher powerful scores and chronotype moderated this relationship. Improved inhibitory control to social reward predicted higher subjective social status in the intermediate and later chronotype group, an effect that was absent in the early group.

**Conclusion:**

This behavioral study found evidence that changes toward a later phase of entrainment predicts social facilitation effects on inhibitory control and higher perceived power among peers. It is proposed here that circadian delayed phase in adolescence is linked to approach‐related motivation, and the social facilitation effects could reflect a social cognitive capacity involved in the drive to achieve social rank.

## Graphical abstract

This paper presents findings that demonstrate that typical changes in circadian timing toward a later phase of entrainment (chronotype) are related to social status outcomes.

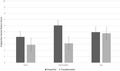

## 1 INTRODUCTION

There are distinct changes in sleep/wake cycles in adolescence whereby the daily rhythms of biological and psychological functions move later within the 24‐hr period and this includes a preference for a sleep onset later in the evening. This phase delay in preference toward “eveningness,” or a shift in “phase of entrainment” toward an evening “chronotype” (Roenneberg, 2015), is driven by pubertal hormones involved in sexual maturation and the transition to adult independence (Carskadon et al., 1993; Hagenauer & Lee, 2012). The onset of endogenous changes in circadian timing, and corresponding alterations in sleep architecture, coincides with the initiation of synaptic pruning (Hagenauer & Lee, 2013), and in parallel with the pubertal maturation of key subcortical brain structures responsible for increased *social motivational tendencies* observed in adolescence (Forbes & Dahl, 2010). This process of *social re‐orientation* (Nelson et al., 2005) manifests in aspects of cognition and behavior that includes increased time spent interacting with peers (Lam et al., 2014), a sensitivity to cues signaling peer acceptance and rejection (Gunther Moor et al., 2010), and greater differentiation between individuals in social status within peer group hierarchies (Koski et al., 2015). Hagenauer and Lee (2012) presented a biobehavioral framework for understanding the influence of the circadian system on reproductive hormones and behavior and hypothesized that the delayed phase was an adaptation in juvenility to the formation and negotiation of social hierarchies. That is, evening time may have afforded opportunities to engage with peers and establish independence away from dominant adults. It is therefore reasonable to hypothesize that circadian phase delay has a role in shaping social neurocognition and could partially explain individual difference variation in social status outcomes. This behavioral study aimed to address this gap by investigating the relationships between chronotype (phase of entrainment) social reward‐related processes and perceived subjective social status in adolescents.

Adolescent research on circadian processes and cognition in healthy populations has largely focused on the impact of the circadian misalignment between biological rhythms and environmental demands (Carskadon et al., 1998). A preference for a later sleep onset collides with earlier school start times, increased extracurricular, and social activities, media use and coincides with relaxation of parental monitoring of bedtimes (Short et al., 2011). Shorter sleep duration, irregular weekday‐weekend sleep patterns, combined with a natural increase in daytime sleepiness observed with pubertal development prove deleterious both for academic learning and for the ability to control emotions and behavior (Crowley et al., 2018; Feinberg & Campbell, 2010).

Current neuroscientific evidence indicates circadian dysregulation and disrupted sleep patterns impact on the protracted development of cognitive functions important for the regulatory control over the heightened exploration, sensation seeking, and impulsivity often observed during adolescence (Hasler & Clark, 2013; Telzer et al., 2015). Given the rapid development of reward‐related sensitivity and approach‐avoidance motivational systems with puberty (Urošević et al., 2012), developmental neuroimaging studies have explored the links between sleep timing and responses to reward cues. Hasler et al. (2012) observed that a greater weekday to weekend shift in adolescents’ sleep patterns was associated with attenuated activity in reward‐related regions as well as prefrontal areas responsible for cognitive control during a monetary reward task. Telzer et al. (2013) reported poorer sleep quality predicted elevated activity in reward‐related areas, lower activity in cognitive control areas, and reduced functional coupling between the two regions in adolescents with greater risk taking tendencies. Longitudinal data suggest irregular sleep patterns reduce white matter tract integrity and potentially the functional integration of neural networks that connect the affective and reward processing subcortical regions with the later developing prefrontal executive control regions, connectivity that underpins behavioral regulation (Telzer et al., 2015).

Adolescent reward systems also engage in response to social cues, attributed to the heightened motivational salience of information that signals peer feedback or status within social hierarchies (Foulkes & Blakemore, 2016; Koski et al., 2015). Imaging studies have observed increased activity in reward processing areas in the anticipation of interacting with socially desirable peers (Gunther Moor et al., 2010; Guyer et al., 2008) and in response to positive social appraisal and peer acceptance compared to children or adults (Gunther Moor et al., 2010; Guyer et al., 2012). Adolescents also demonstrate heightened reactions to negative social evaluation, peer rejection (Burnett et al., 2011; Silk et al., 2012), and social exclusion (Sebastian et al., 2010). The level of neural activity in affective and cognitive control regions can also vary dependent on the relative social status of the respondent, with greater engagement observed in high status participants only when faced with the potential of social exclusion by similarly high‐status peers (de Water et al., 2017). Information about relative social status is processed within reward‐related neural networks (Saxe & Haushofer, 2008), and the potential to improve one's social status is in and of itself a potent social reward (Cardoos et al., 2017). Investigations into the neurobehavioral mechanisms involved in social dominance traits also focus on individual differences in the regulatory control of responses to reward cues (Lozano‐Montes et al., 2019). In summary, there is a sound theoretical basis from which to predict that circadian timing and sleep patterns are implicated in the development of both social and nonsocial reward processing mechanisms and that inhibitory control in response to reward‐related cues is an appropriate neurocognitive measure to validate any link between chronotype and social status outcomes.

There is a well‐documented association between a later evening phase preference and increased risk for poorer academic, mental health, and social adjustment outcomes (Randler, 2011). This is attributed to a shared genetic vulnerability to both circadian disruptions and neuropsychiatric disorders, further compounded by environmental factors (Taylor and Hasler 2018). It is proposed the circadian system orchestrates the biological processes that underpin motivated behaviors for eating, sleeping, and mating (Antle & Silver, 2015). There is also an evolutionary psychology perspective that suggests circadian phase delay is likely related to the greater sexual activities afforded at night‐time, and this would include motivated behaviors toward increased social status (Ellis et al., 2012). Social hierarchies are pervasive structures and seen as a key organizational principle of cognition and behavior from early childhood (Koski et al., 2015). Hierarchies are multidimensional and exist across numerous different domains (i.e., physical attractiveness, academic achievement, popularity) and a typical adolescent may occupy different positions across dimensions (Sweeting et al., 2011). In the early to mid‐adolescence period, a higher social status among peers is prioritized above other domains that include academic achievement, adherence to rules, friendships and empathy toward less fortunate others (LaFontana & Cillessen, 2010). As peer acceptance or rejection predicts heightened reward‐related activations and social status outcomes, it is an appropriate context in which to investigate the link between chronotype and social hierarchies.

A relationship between social reward and social status outcomes is expected based on prior research. The effects of chronotype on social reward and social status outcomes have not been previously assessed directly. The indirect evidence reviewed above suggests a later chronotype might predict poorer reward‐related inhibitory control and lower social status. This dysfunction hypothesis is based on the association observed between psychological problems in those with a later chronotype (Gariépy et al., 2019; Randler, 2011), that greater problems are seen with lower social status (Rivenbark et al., 2019) and that circadian misalignment and poor sleep quality that are more often observed with a later phase, predict weakened reward‐related cognitive control (Hasler & Clark, 2013; Telzer et al., 2013). It is unclear therefore whether effects of chronotype on social cognition and social status exist, and if they remain independent of disrupted sleep patterns or the presence of behavioral difficulties.

### 1.1 Current study

The conceptual framework proposed here adopts the view that the change in phase of entrainment in typical adolescence reflects maturation of a trait related to social functioning. It is predicted that effects of chronotype will be observed in cognition (reward processing) and in behavior (social status). It is also predicted that social reward (compared with nonsocial) processing will better predict social status outcomes. If predictions are confirmed two distinct and equally plausible exploratory models can be tested (a) that reward‐related inhibitory control is a mediator of chronotype and social status and (b) chronotype is a moderator of reward‐related inhibitory control and social status outcomes. The first model assumes that any relationship between chronotype and social status is explained by cognitive responses to reward contexts. The second model assumes the association between reward processing, and social status can vary along the dimension of chronotype.

This study focused on a limited age range in mid‐adolescence based on both circadian and behavioral observations. The mid‐adolescence period marks approximately the onset of the steepest slope in phase delay that peaks, on average, at aged 18 in females and at 19 in males (Fischer et al., 2017). In this period, there is also an observed peak in socially oriented behaviors and in peer influence (Sebastian et al., 2010; Steinberg & Monahan, 2007) as well as an increasing sophistication in social cognitive capacities (Crone & Dahl, 2012). This may therefore be a key period in the development of cognition related to social differentiation. To study this age range in the first instance may also minimize the potential impact of the greater circadian misalignment observed in later adolescence.

## 2 METHODS

### 2.1 Participants

A total of 75 adolescents aged 13–14 years (mean = 13.6, standard deviation = 0.39) participated in the study. The sample consisted of 41 (54.7%) females. Participants attended a mainstream state secondary school in an area ranked 17.215 out of 32,844 in the Indices of Deprivation, England 2019. At the request of the school, there was no inclusion and exclusion criteria and all students in the year group were eligible and invited to participate. Participants were 96% White British with English as a first language that is consistent with the low ethnic diversity seen at a rural regional level (UK department for Environment FRA, 2020). G*Power (Faul et al., 2009) was used to perform a sample size calculation for a linear multiple regression with three predictors based on an estimate of a moderate effect size (partial f^2^ = 0.15) with a power of 0.80 at an alpha level set at 0.05. This resulted in a total sample size of *N* = 77. The study was completed in the winter months December to February. Participants completed a 40‐min individual assessment session that was comprised of an eye tracking task and anonymized computerized self‐report questionnaires. The order of completion of the questionnaires and eye tracking task were counterbalanced across participants.

### 2.2 Materials and procedure

#### 2.2.1 Chronotype

Participants completed the English full version of the Munich Chronotype Questionnaire (MCTQ) (Roenneberg et al., 2003) for children and adolescents. The measure of chronotype is based on the of timing of weekend midsleep and has been used extensively to investigate the relationship between sleep timing, adolescent behavior and development (Gariépy et al., 2019). It has been validated against objective measures including actigraphy (Santisteban et al., 2018) and dim light melatonin onset (Kantermann et al., 2015) and is a reliable self‐report instrument to estimate phase of entrainment. The MCTQ measures phase of entrainment in time and operationalizes chronotype as a continuous trait. Weekend midsleep is calculated as the midpoint between sleep onset and offset on school free days, corrected for sleep debt accumulated during school days (MSFsc). The weekday and weekend duration and the relative social jet lag (calculated as discrepancy between midsleep on weekdays and weekends) were selected as covariates to test for effects of shorter sleep and circadian misalignment (Wittmann et al., 2006).

#### 2.2.2 Subjective Social Status (SSS)

Social status among peers was measured using the SSS‐school dimensions (Sweeting et al., 2011) that are adapted from MacArthur SSS scale for adolescents (E. Goodman et al., 2001). The scale asked how popular, powerful, attractive, respected, sporty, doing well at school, or a troublemaker they are compared with the rest of the year group (not only their friendship group) on a scale of 1–10 (1 is highest). Scores were reverse coded so that a higher score indicate higher subjective social status. The SSS‐school dimension scales show internal consistency and have been externally validated. A principal component analysis on responses from adolescents (*N* = 3,194) found all scales load onto “SSS‐Peer” domain (loadings range 0.261–0.829 with 47% variance explained). Higher scores were predicted by more friendship nominations (ηp^2^ = 0.027) and higher interviewer‐rated attractiveness scores (ηp^2^ = 0.020) (Sweeting et al., 2011).

### 2.3 Covariates

The study selected measures of pubertal timing, sleep‐related factors, social media use, and behavioral difficulties to include as covariates based on past research literature. The Pubertal Development Scale (PDS) is a validated self‐report measure of physical development and was included to account for influence of puberty on social status (Teunissen et al., 2011). The pubertal development score was calculated for male and female participants based on the published instructions (Petersen et al., 1988). Additional validated instruments were included that assessed levels of daytime sleepiness, screen‐based media use, and behavioral problems. The Cleveland Adolescent Sleepiness Questionnaire (CASQ) (Spilsbury et al., 2007) is a validated age appropriate measure of sleep‐related impairment (Hanish et al., 2017) and is comprised of 16 items probing experiences of sleepiness and alertness throughout the day, both at school and at home. A measure of screen‐based media (SBM) included four items from the large scale UK Household Longitudinal Study (Booker et al., 2015) that probed the number of hours on a normal school day spent on screen‐based media and while in bed. Participants were also screened for behavioral difficulties using the Strengths and Difficulties Questionnaire (SDQ) for ages 4 –17 years (R. Goodman, 2001). The SDQ has been shown to have good concurrent validity (Muris et al., 2003) and has been extensively used in clinical and research settings.

### 2.4 Eye movement experimental paradigm

It is unknown whether chronotype modulates social versus nonsocial incentivized processing and therefore a paradigm that probes responses to the two reward types was employed. Two highly replicated but structurally different reward contexts were interleaved with blocks of classic antisaccades. This was so that the context varied but inhibitory control performance was measured under identical task conditions.

#### 2.4.1 Social reward context

The Cyberball Game (Williams & Jarvis, 2006) was used as an experimental manipulation of social reward context. It is a classic experimental paradigm shown to engage neurocognitive architecture associated with social incentive motivation in adolescents (Kray et al., 2018). Cyberball is an online ball throwing game whereby participants are told they are playing against peers over a computer network. Participants are informed the game tests the effects of mental visualization on performance. The crucial manipulation is the number of times the ball is thrown to the participant. The task is not in fact networked, and trials are systematically controlled by the experimenter. In order to create a needs threat of social exclusion the ball is thrown fewer times toward the participant compared with other players. This study employed Cyberball version 5.0 with two anonymous players of unknown gender. The task included a short demonstration to familiarize the participant with the task. The experiment included one inclusion block that contained 66% inclusion throws from a total of 12 throws (participant included every 3rd ball thrown) and one exclusion block of 66% exclusion throws (participant included 2nd and 5th ball thrown only). The Cyberball instructions were displayed on the computer screen and read aloud by the experimenter. Participants subsequently completed the Needs Threat Questionnaire (NTQ) administered with this task (Williams et al., 2000). It measures needs threat using four different constructs; belonging, control, self‐esteem, meaningful existence. All participants completed the NTQ after the Cyberball task.

#### 2.4.2 NonSocial reward context

The nonsocial reward context used in the study was the Card Guessing Game and has been previously described (Nusslock et al., 2012). The game involves presenting a card with a question mark. Participants are asked to guess whether the flip side to the card will show a number greater or less than five. There are two types of trial and trial type is indicated after the guess has been entered. A win trial is indicated by an upward arrow and a loss trial by a downward arrow. Participants are awarded 50 points for correct guesses on win trials and penalized 50 points for incorrect guesses on loss trials. Trial outcome was indicated by a green upward arrow for a win, a red downward arrow for a loss and a yellow circle for no change in points. Trial outcomes was controlled by the experimenter. Participants completed a practice block of 7 trials with each step manually controlled by the experimenter, and participants needed to demonstrate they understood the task instructions prior to beginning of the experimental blocks of 12 trials. A win block contained 66% win trials and a loss block contained 66% loss trials.

The order of presentation of the social and nonsocial reward contexts was counterbalanced across participants. All participants were tested between 9 a.m. and 1 p.m. to minimize the effects of time of day at testing on cognitive performance (Hahn et al., 2012).

#### 2.4.3 Antisaccade inhibitory control task

The use of an antisaccade task to test effects of context on inhibitory control has been used previously with a card task (Jazbec et al., 2006) and with the Cyberball paradigm (Jamieson et al., 2010). The antisaccade task required participants to inhibit an automatic response to look toward a target and instead look to the opposite side of the screen. An Eyelink 1,000 Desktop Mount eye tracker (SR Research) at a monocular sampling rate of 1,000 Hz was used, and the experiment was programmed and run with Experiment Builder software (SR Research). Participants sat 60 cm from a flat 19‐inch LCD monitor (60 Hz refresh rate) with a desktop mounted chin rest. Participants completed a practice block of 20 prosaccades (looking toward the target) and 20 antisaccades (looking away from the target) prior to introduction to the first experimental context. Participants were required to complete a total of four blocks of 20 antisaccades trials. Two within the social reward context after the inclusion block and the exclusion block and two within the nonsocial reward context after the win block and after the loss block. The order of context and block type were counterbalanced. Participants were given standard instructions for the antisaccade task and told it was a measure of attention unrelated to performance on the Card Guessing and Cyberball contexts. No further information was provided on aspects of the experimental design.

### 2.5 Ethics

The research was approved by University Research Ethics committees and conducted in accordance with the World Medical Association Declaration of Helsinki. Parents provided informed written consent and adolescents informed assent prior to participation.

### 2.6 Eye tracking data processing

Processing of the antisaccade task data found an average of 87.6% valid trials available for analyses. The number of valid trials varied across individual participants, but no systematic differences existed between trial blocks or context conditions. Valid trials were comprised of on average, 32% error saccades, typical of performance in previous adolescent research (Hardin et al., 2007). Missing data included no value for midsleep on free days for 6 children as they were unable to report time of sleep offset on a weekend and were therefore excluded, resulting in a total sample of 69 participants available for the final analyses.

### 2.7 Statistical analyses

The data from the experimental task were analyzed first to test (1) the relationship between phase preference and reward‐related response inhibition using two‐level random intercept multilevel generalized linear mixed‐effects model (GLMM). The design of the experimental task has a hierarchical structure with trials nested within blocks nested within participants. The performance metric of interest was the binary outcome at the trial level of a correct antisaccade or an error prosaccade, as a measure of inhibitory control. The level 1 data were specified as repeated measures of trials nested within blocks. An autoregressive AR (1) covariance structure was selected as is appropriate for time series data. A random intercept was specified for participant with a scaled identity random covariance type. Chronotype when measured by the MTCQ is a dimensional construct and measured on a continuous scale. The MTCQ midsleep scores were not a significant linear predictor of saccade performance. Curve estimation of saccade error probabilities and MTCQ midsleep scores showed a linear fit was nonsignificant (*r* ^2^ = .001, *F* (1, 4,834) = 2.43, *p* = .119) and an inverse equation was the best fitting model (*r* ^2^ = .004, *F* (1, 4,834), *p* < .001). MCTQ Midsleep scores was divided into terciles to represent early, intermediate, or late chronotypes, consistent with prior research (Gariépy et al., 2019) and appropriate when the study population is from the same geographical region and assessed at the same time of year (Roenneberg, 2015). There were no differences in performance during the practice block of 20 antisaccade trials across the three chronotype groups. The statistical analyses of the experimental task included a main effect of chronotype (early, intermediate, late) and a main effect of Context (Cyberball, Card Guessing) and the chronotype × Context interaction term. Preliminary model building involved testing for order effects of context presentation (i.e., Cyberball or Card Guessing first), task block type (win or loss / accept or reject), or task block valence (win or accept / loss or reject). Chronological age was included in the model to account for age‐related improvement in antisaccade performance. Note that no effects of the NTQ scales were found (see Table S1). The alpha level in all comparisons between more than two groups in the study was Bonferroni adjusted. To test whether any effects remained after accounting for pubertal, sleep, and behavioral factors, the pubertal scale scores, sleep‐related variables (duration, social jet lag, daytime sleepiness), social media use, and behavioral difficulties were entered as covariates.

To assess (2) the relationship between chronotype and social status a Repeated Measures General Linear Model (GLM) tested for effects of chronotype group on the scores of the seven SSS subscales. To test (3) the relationship between reward response inhibition and Social Status participants’ mean error probabilities, calculated from the parameter estimates derived from the GLMM, were entered as predictors in Repeated Measures GLM on the seven Social Status scores. To determine (4) whether reward processing mediated chronotype and social status a mediation analyses was performed using Hayes Process for SPSS. Unstandardized indirect effects were computed for each of 5,000 bootstrapped samples, and the 95% confidence interval. To test whether chronotype was a moderator of reward response inhibition and social status a moderation analyses was performed using Hayes Process for SPSS. All models were checked for violation of assumptions. Checks for outliers prior to moderation analysis identified 2 cases from the early group with a Cook's D value above the recommended 4/n for error probabilities. The two scores were not due to measurement error and were below 2.5 *SD*s of the distribution. Removal of the values did not alter the nonsignificant relationship between predictor and outcome in this group, and the values were retained in the final model. All raw data and syntax for the analyses are available at https://dataverse.harvard.edu. All statistical analyses were performed in SPSS version 21.0 (IBM Corp).

## 3 RESULTS

### 3.1 Chronotype and reward‐related response inhibition

**FIGURE 1:**
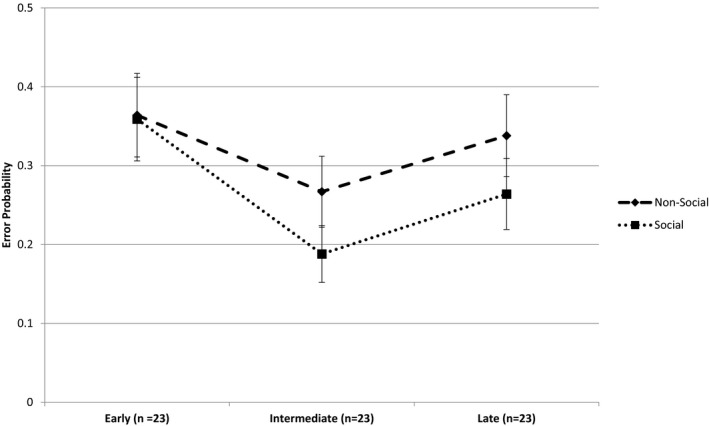
Mean (SE) predicted antisaccade error probabilities for each Chronotype tercile group in the Social (Cyberball) and Non‐social (Card Guessing) contexts

The results of the GLMM on antisaccade reward task performance are presented in Table 1. There was a significant main effect of Context with a higher probability of errors in the nonsocial context (*M* = 0.321, *SE* = 0.029) compared with the social context (*M* = 0.264, *SE* = 0.026). There was no significant main effect of chronotype on overall error probabilities whereas the chronotype × Context Interaction was significant. The mean (*SE*) error probabilities for the early, intermediate and late chronotypes within the two different contexts are displayed in Figure 1. Pairwise contrasts within each chronotype showed no difference in error probabilities in early chronotypes (contrast = −0.005, *SE* = 0.026, *t* = 0.179, *p = *.858) whereas intermediate chronotypes showed a significantly lower likelihood of errors in the social compared with the nonsocial context (contrast = −0.079, *SE* = 0.024, *t* = −3.309, *p = *.001), as did the Late chronotypes (contrast = −0.074, *SE* = 0.027, *t* = −2.813, *p = *.005). In summary, the intermediate and late chronotype groups showed improved antisaccade performance in the social reward (Cyberball) compared with the nonsocial (Card Guessing) reward context. An effect that was absent in the early group. An overall reduction in error probability (improved performance) was also associated with an increase in chronological age (*b* = −0.821, *SE* = 0.347, *t* = −2.365, *p = *.018) and increase in the number blocks performed from 1 to 4 (*b* = −0.139, *SE* = 0.031, *t* = −4.448, *p < *.001). There was a trend for participants to show an increased likelihood of error on trials that followed a task block with a negative valence (loss or exclusion trials) compared with positive valence (win or inclusion trials) independent of Context (contrast = −0.027, *SE* = 0.014, *t* = 1.915, *p = *.056). This was not qualified by any interactions.

**TABLE 1:** Fixed and random effects on the probability of performing a prosaccade error in the antisaccade task

	*F* value	*p* value	
Fixed Effects (*df*)			
Context (1)	15.55	<.001	
Chronotype (2)	2.25	.105	
Chronotype × Context (2)	3.68	.025	
Age (1)	5.59	.018	
Block Valence (1)	3.70	.055	
Block Number (1)	6.91	<.001	

Random Effects	Estimate (*SE*)	Z	*p* value [95% CI]
Participant	1.054 (0.211)	4.999	<.001 [.712, 1.559]
Residual			
AR1 Diagonal	0.956 (0.020)	48.623	<.001 [.919, 0.996]
AR1 Rho	0.036 (0.016)	2.310	.021 [.005, 0.066]

The measures of puberty, social jetlag, weekend and weekday sleep duration, time of testing, daytime sleepiness, screen‐based media, and total behavioral problems were entered into the model. An increase in error probabilities was significantly predicted by a lower PDS score (less physical development, *p* = .008), a shorter weekend sleep duration (*p* = .05), and higher daytime sleepiness as measured by CASQ total score (*p* = .044). No other significant effects were found, and the addition of the covariates did not modify the results reported in Table 1. Means (*SD*) of the puberty, sleep, and behavioral measures for each chronotype group are presented in Table S1 and the results of the model that included these covariates are reported in Table S2.

### 3.2 Chronotype and social status

**FIGURE 2:**
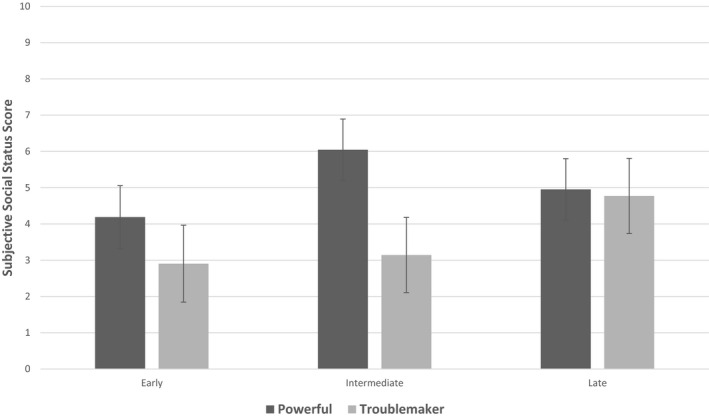
Mean (95% CIs) subjective scores between 0 and 10 (10 is high) for each Chronotype tercile group for the Powerful and Troublemaker Subjective Social Status subscales

The Repeated Measures GLM to test the effect of chronotype on the seven SSS subscales found no main effect of chronotype *F* = (2,62) 1.746, *p* = .183, η_p_^2^ = 0.053. There was a significant chronotype × Subscale interaction *F* = (7.87, 243.88) 2.594, *p* = .003, η_p_^2^ = 0.077. Follow‐up Bonferroni adjusted post hoc comparisons found an effect of chronotype for the SSS subscale “Powerful.” Pairwise contrasts showed intermediate chronotypes reported occupying a more powerful social status position among peers compared with that reported by early chronotypes (contrast = 1.857, *SE* = 0.606, *p* = .010). There was also an effect of chronotype on the subscale “Troublemaker.” The late chronotypes reported being greater troublemakers than early chronotypes (contrast = 1.834, *SE* = 0.720, *p* = .040). The means (*SE*) of the two scales are displayed in Figure 2. To check whether the effect of chronotype was not better accounted for by reported behavioral difficulties, a repeated measures GLM was conducted on SDQ subscales. The analysis found no main effect of chronotype *F* = (2,66) 1.283, *p* = .284, η_p_^2^ = 0.039 and no significant chronotype × Subscale interaction *F* = (8, 252) 0.967, *p* = .462, η_p_^2^ = 0.030. The means (*SD*) of the SDQ scales by chronotype group are reported in Table S1.

### 3.3 Reward‐related response inhibition and social status

The Social context mean error probabilities were included as a predictor in a repeated measures GLM of the Powerful and Troublemaker subscale scores. The analysis found no overall main effect of errors *F* = (1,67) 1.42, *p* = .237, η_p_^2^ = 0.021 whereas the subscale × errors interaction was significant *F* = (1, 67) 4.79, *p=*.034, η_p_^2^ = 0.066. The parameter estimates indicated lower error probabilities in the Cyberball Context predicted higher Powerful subscale scores *b* = −3.065, *SE* = 1.23, *t* = −2.486, *p* = .015 η_p_^2^ = 0.084 and no relationship with Troublemaker scores *b* = 0.29, *SE* = 1.54, *t* = 0.287, *p* = .852 η_p_^2^ = 0.001. The GLM with nonsocial error probabilities as a predictor found no main effect of errors *F* = (1,67) 0.35, *p* = .558, η_p_^2^ = 0.005 and no subscale × errors significant interaction *F* = (1,67) 2.70, *p* = .105, η_p_^2^ = 0.039. A difference score calculated from Social minus nonsocial error probabilities was a significant predictor of higher Powerful scores *R* ^2^ = .065, *F* (1, 67) = 4.44, *p* =.039.

### 3.4 Chronotype, reward, and social status

The results above show that chronotype and social error probabilities predict Powerful social status scores. A simple mediation analysis tested whether chronotype and Powerful status scores were mediated by social reward error probabilities. Troublemaker scores were added as a covariate. The total effects model showed the coefficient for social error probabilities on Powerful scores was significant *b* = −2.37, *t*(64) −2.02, *p* = .048, and the coefficient for the intermediate chronotype group on Powerful scores *b* = 1.52, *t*(65) 2.83, *p* = .006 remained significant. The indirect effect therefore of chronotype group on Powerful scores was not significant with an effect size = 0.03, *SE* = 0.21, 95% CI [−0.03,.81] indicating no mediation effect. These results were independent of the relationship between Troublemaker and Powerful scores *b* = 0.34, *t*(65) 3.60, *p* = .0006.

**FIGURE 3:**
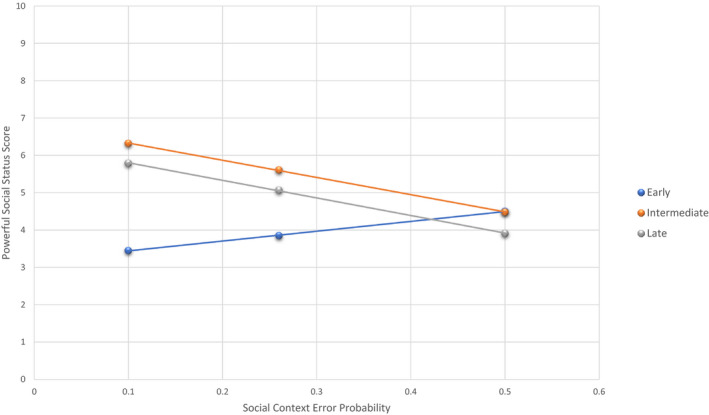
The simple slopes for social error probabilities predicting Powerful scores at below 1SD, mean and above 1SD for each Chronotype Group

A moderation analysis tested whether the magnitude of the relationship between social error probabilities and Powerful scores varied for different chronotypes. Social error probabilities, chronotype, and Troublemaker scores as a covariate accounted for a significant amount of variance in the powerful scores *R* ^2^ = .37, *F* (6, 62) = 6.00, *p* = .001. The addition of the social error probabilities and chronotype group interaction term explained a significant increase in variance in Powerful scores Δ*R* ^2^ = 0.07, *F*(2,62) = 3.41, *p* = .040. This indicates evidence of moderation with a small effect size. The simple slope coefficient was not significant in the early group *b* = 1.67, *t*(62) 0.86, *p* = .40 and was significant in the intermediate *b* = −5.47, *t*(62) −2.29, *p* = .026 and Late group *b* = −3.91, *t*(62) −2.29, *p* = .024. The results show that lower social error probabilities predict higher reported Powerful social status in intermediate and late chronotypes, whereas this relationship was absent the early chronotype group. Figure 3 displays the simple slopes for social error probabilities predicting Powerful scores as −1 *SD*, μ, + 1 *SD* for each chronotype group.

## 4 DISCUSSION

The study found evidence to support the hypothesized relationship between circadian phase of entrainment, social cognitive control, and subjective social status outcomes in typically developing adolescents. The effects of chronotype on cognition and social status showed a consistent pattern. An intermediate and late delay phase of entrainment predicted improved inhibitory control in a social reward context that related to higher perceived powerful status compared with peers. Although the effect of social reward context on cognitive control was the most pronounced in the intermediate chronotype group, the moderation analysis confirmed social reward‐related inhibitory control predicted higher reported powerful subjective status in both intermediate and late chronotypes. This is preliminary evidence that chronotype is an individual differences trait associated with modulation of the processing of social reward cues that are relevant for the achievement of social status.

There was limited support for the dysfunction hypothesis that a later chronotype would predict weakened inhibitory control and lower social status. There was however greater variability in cognitive and social status outcomes in this group. A later chronotype also predicted higher “Troublemaker” social status scores but this was unrelated to reward‐related inhibitory control in this study. The potential reasons for this are discussed in more detail below.

The ability to perform a correct antisaccade involves top down signals from the prefrontal cortex onto subcortical sensorimotor networks in order to initiate and maintain a consistent neural “task set” for volitional control of the eye movement (Everling & Johnston, 2013; Hwang et al., 2010). The improved AS performance observed in the Cyberball context therefore reflects increased attentional engagement in response to presence of social cues. This is consistent with classic social facilitation effects, whereby the mere presence of others narrows attentional focus and results in improved performance on simple cognitive tasks (Sharma et al., 2010; Wolf et al., 2015). Research on social facilitation (or audience effects) in adolescents report performance decrements on complex tasks (Wolf et al., 2015), whereas in adults, facilitation effects in simple AS tasks have been found on processing speed but not on inhibitory control (Oliva et al., 2017; Tricoche et al., 2020). It would therefore appear social facilitation effects depend on task complexity and involve speed‐accuracy trade‐offs that undergo maturational changes from adolescence to adulthood. Social facilitation effects on performance are attributed to increased activity in approach‐related motivational systems (Schnuerch & Pfattheicher, 2018). Prior research has largely focused on the role of circadian misalignment in disruptions to approach motivational systems implicated in addictive and affective disorders (Hasler et al., 2010; Hasler & Clark, 2013). This is the first known report of a link between adolescent chronotype and social facilitation effects on cognitive performance in typically developing adolescents.

It is important to recognize that social and nonsocial reward processing involves largely overlapping neural architecture, the difference in behavioral outcomes by reward type more likely reflect differences in the motivational value attached to incentives in any given context (Rademacher et al., 2010). Studies that have compared directly social and nonsocial reward responses report that adolescents rated social rewards as more subjectively motivating than monetary rewards (Wang et al., 2017). In the same paradigm, electrophysiological signals of response speed and cognitive control correlated only under conditions of social reward, interpreted as increased sensitivity and attentional engagement to social reward cues in the adolescent group (Wang et al., 2020). Longitudinal neuroimaging research on effects of incentives on inhibitory control show substantial individual differences in terms of whether incentives will improve or impair performance, and the brain circuitry underpinning performance is shown to follow a protracted developmental trajectory into adulthood (Paulsen et al., 2015). The findings here provide a signpost the potential role of the circadian system in both typical maturational changes and individual differences in the development of incentive processing.

The observation that social facilitation related to power subscale of subjective social status is also consistent with social psychological research on the effects of power on cognition (Cho & Keltner, 2020). The approach‐inhibition theory posits that elevated power activates approach‐related tendencies that include increased attention toward rewards, and a greater ability to focus attention on the relevant aspects of task demands. Powerful individuals also exhibit disinhibited behavior, so a clearer delineation between social facilitation effects and the different components of social dominance is warranted. Developmental research on the behavioral characteristics associated with social dominance describes individuals with the highest overall adjustment and peer acceptance as socially “bistrategic” (Hawley, 2007). That is, effective social influence involves both prosocial as well as moderate levels of disruptive or deviant behaviors (Jonkmann et al., 2009). The lack of association between social reward‐related responses and the elevated troublemaker scores in later chronotypes would indicate social facilitation effects may be a marker of the positive social adjustment components of social dominance.

In respect to understanding longer term outcomes, a meta‐analysis of individual differences adult research on social facilitation indicates two general orientations in response to the social presence of others; positive‐assured and negative‐apprehensive. Individuals with a positive orientation are self‐assured, report higher self‐esteem, and greater extraversion (Uziel, 2007), and, albeit an indirect link, evening types high on extraversion also report greater life satisfaction compared with evening types high on introversion (Drezno et al., 2019). Circadian‐related disruption to the appetitive/approach motivational system is also implicated in the increased risk of affective and addictive disorders in those with an evening preference (Hasler et al., 2010; Hasler & Clark, 2013). Social facilitation effects could be an informative metric used to investigate the neurobehavioral mechanisms that link circadian rhythms to personality dimensions or psychopathology.

It is also important to note that chronotype effects on inhibitory control remained independent of variance explained by age, pubertal development, and the negative impact of daytime sleepiness. This provides further support for the interpretation that chronotype is a potential moderator of social cognition and behavior. This hypothesis requires to be investigated further using direct assessments of the relevant constructs, including measures of social dominance traits and social status hierarchies using peer nomination techniques. Overall, the findings suggest a potential role of delayed phase of entrainment in the activation of social approach motivations that include the drive to achieve social dominance or improved social rank in typical adolescent development.

### 4.1 Limitations

This behavioral study did not adopt biological measures of circadian phase delay based on salivary melatonin secretion or on core body temperature to validate the self‐report chronotype measure. As chronotype was determined from the sample's midsleep distribution, it is important to compare the sample characteristics to larger population research with the same instrument. The average weekend midsleep values in the intermediate chronotype observed here closely approximate the total sample mean value for 15‐year olds reported by Fischer et al., (2015), whereas our sample had fewer with extreme late values (> 6:00). Prior research with the SDQ has also reported increased behavioral and social adjustment problems in adolescents with an eveningness phase preference (Lange & Randler, 2011). This prior study included older adolescents and a larger age range, reported difficulties increase with age, and used the Morningness Eveningness Questionnaire (Horne & Östberg, 1976) as opposed to the MCTQ. It may be however that adolescents with an extreme phase delay or more severe adjustment difficulties were underrepresented in the present sample. It is also recognized that the present sample were not representative in terms of ethnic diversity at an urban or national level or can address the ethnic differences found in chronotype in adults (Malone et al., 2016).

It is important to acknowledge that subgroups may exist along the phase delay spectrum that differ in risk for adverse outcomes. Social and academic adjustment has been found to be similar to morning types when evening types report being good sleepers (Tavernier & Willoughby, 2014). We noted a shorter sleep duration in the evening chronotype group (Table S1), and although this did not alter the outcomes of the study, the presence of sleep difficulties is a stronger predictor of negative social adjustment trajectories than chronotype alone (Jiskrova et al., 2019). No gender effects emerged as the task contexts may have not have sufficiently probed gender‐related cognition as no information was provided about the gender of the anonymous “players” in the Cyberball task. Further research will need to explore if social facilitation effects vary by gender and in what contexts, given the known gender differences in reward sensitivity, sensation seeking and impulse control in younger adolescents (Wang et al., 2017).

The trend for greater errors subsequent to loss or exclusion suggests a negative valence weakened cognitive control independent of context, which is partially consistent with past research. Prior studies with monetary reward AS tasks have report improved performance in loss versus gain trials (Jazbec et al., 2006) and in valence versus neutral conditions (Hardin et al., 2007). The present study design did not permit analyses of stages of reward processing (i.e., anticipation, receipt, or evaluation) that may have provided insights in terms of impact of valence on performance. Research using the Cyberball Game has reported increased AS errors after experience of exclusion (Jamieson et al., 2010), and more recently exclusion predicted impaired performance on a N‐back task in a sample of 12–14 year olds adolescent females (Fuhrmann et al., 2019). This indicates ostracism impacts negatively on inhibitory and working memory components of executive function. The differences between prior research and the present study therefore are likely attributable to the inclusion in prior research of trial level performance feedback that can increase the salience of potential loss and that valence and neutral trials were collapsed within blocks in the present study. No evidence was found that effects of valence on performance varied by chronotype and facilitated performance with a positive valence is consistent with the approach motivational account outlined above. There is also the limitation that self‐reported subjective perception of greater “powerfulness” is not a validated measure of a social dominance trait. It is not yet clear if these findings will translate to real world settings.

## 5 CONCLUSION

This work has contributed to our understanding of the role of the circadian system in adolescent social development. Typical changes in adolescent circadian timing appear related to neurocognitive processes consistent with approach motivations, and a moderate shift in timing is more consistently associated with perceived social power among peers. This is a cross‐sectional behavioral study, and developmental changes require investigation with the same individuals over time. Clearer hypotheses can now be proposed in regard to elucidating the link between circadian phase of entrainment on social facilitation effects. This includes endocrinological and physiological markers of circadian rhythmicity and social cognition, and measurement of diurnal variations in social facilitation effects across the 24‐hr period. The inverse relationship observed between chronotype and both cognition and behavior also indicate that comparisons based on morningness vs. eveningness types will be less informative. Theoretical models on social facilitation in adolescent development should consider circadian delayed phase of entrainment as a potential marker of approach motivational tendencies toward social dominance.

## 6 ETHICAL PUBLICATION STATEMENT

We confirm that we have read the Journal's position on issues involved in ethical publication and affirm that this report is consistent with those guidelines.

## CONFLICT OF INTEREST

There are no conflicts of interest to declare.

## AUTHOR CONTRIBUTIONS

J.L, T.D. and G.A.P conceived of the presented idea. J.L and P.M developed the theory. J.L, T.D, T.W, and G.A.P developed the eye tracking paradigm. J.L and T.D collected the data. T.W pre‐processed the eye tracking data. J.L conducted and F.D verified the statistical analyses. All authors reviewed the results and contributed to the final manuscript.

### Peer Review

The peer review history for this article is available at https://publons.com/publon/10.1002/brb3.2090.

## Supporting information

Table S1‐S2

## Data Availability

The data that support the findings of this study are openly available in Harvard Dataverse at https://doi.org/10.7910/DVN/F4EK7F.
