# Supplementary material for: The role of chronotype and reward processing in understanding social hierarchies in adolescence
Source: Brain Behav. 2021 Mar 1;11(5):e02090. doi: 10.1002/brb3.2090 (PMC8119846; doi:10.1002/brb3.2090)
Supplement: Supplementary file 1 — Table S1‐S2 [file BRB3-11-e02090-s001.docx]

Table S1

Participant characteristics and descriptive statistics for the study measures by chronotype

| N = 69 | Early Chronotype  (n = 23) | Intermediate Chronotype  (n = 23) | Late  Chronotype  (n = 23) |
| --- | --- | --- | --- |
| Male (%) | 10 (43.5%) | 10 (43.5%) | 10 (43.5%) |
| Age in years (SD) | 11.1 (2.3) | 11.9 (2.4) | 10.5 (2.6) |
| Time at Test (SD) | 10.9 (1.1) | 11.0 (1.2) | 11.2 (1.3) |
| Baseline Errors (SD) | 0.38 (.05) | 0.31 (.05) | 0.47 (.06) |
| PDS (SD) | 2.6 (.75) | 2.7 (.62) | 2.9 (.50) |
| SBM hours (SD) | 8.0 (1.7) | 8.4 (1.1) | 8.5 (1.7) |
| CASQ Total (SD) | 30.8 (8.5) | 31.9 (6.1) | 33.7 (7.1) |
| MCTQ |  |  |  |
| MSF (SD) | 3.4 (.83) | 4.4 (.84) | 5.3 (1.1) |
| Weekday Duration (SD) | 7.74 (1.5) | 8.21 (.93) | 7.99 (1.8) |
| Weekend Duration (SD) | 10.2 (1.5) | 9.8 (1.3) | 8.3 (1.4) |
| Relative Social Jetlag | .42 (.91) | 1.27 (.76) | 1.77 (1.2) |
| NTQ |  |  |  |
| Belonging | 3.2 (.93) | 3.4 (.87) | 3.4 (1.0) |
| Control | 3.8 (.95) | 3.9 (.91) | 3.7 (1.1) |
| Self Esteem | 3.5 (1.1) | 3.9 (.96) | 3.6 (1.1) |
| Meaningful Existence | 4.1 (.91) | 3.8 (.97) | 4.1 (1.3) |

Table S1 Continued

| N = 69 | Early Chronotype  (*n* = 23) | Intermediate Chronotype  (*n* = 23) | Late  Chronotype  (*n* = 23) |
| --- | --- | --- | --- |
| SSS |  |  |  |
| Popular (SD) | 5.7 (2.1) | 6.5 (1.9) | 6.5 (2.4) |
| Academic (SD) | 6.8 (2.0) | 6.9 (2.2) | 5.7 (2.2) |
| Powerful (SD) | 4.2 (1.9) | 5.8 (1.8) | 4.9 (2.2) |
| Troublemaker (SD) | 3.0 (2.0) | 3.3 (2.1) | 4.7 (2.9) |
| Attractive (SD) | 5.0 (2.3) | 6.1 (2.1) | 5.0 (2.4) |
| Respected (SD) | 5.5 (2.1) | 6.6 (1.8) | 6.2 (2.3) |
| Sporty (SD) | 6.2 (3.1) | 6.9 (2.6) | 5.7 (3.2) |
| SDQ |  |  |  |
| Emotional Problems (SD) | 3.9 (2.4) | 4.0 (2.0) | 4.1 (3.0) |
| Conduct Problems (SD) | 1.4 (1.3) | 2.1 (1.4) | 2.5 (1.7) |
| Hyperactivity (SD) | 4.0 (2.4) | 4.5 (2.1) | 5.1 (1.7) |
| Peer Problems (SD) | 2.0 (1.7) | 1.6 (1.4) | 2.2 (2.1) |
| Prosocial (SD) | 7.6 (1.4) | 7.3 (1.5) | 7.5 (1.2) |
| Total Difficulties (SD) | 11.6 (5.6) | 12.2 (4.2) | 13.9 (5.2) |

Table S2. Fixed and random effects on the probability of performing a prosaccade error in the antisaccade task including developmental, sleep and behaviour covariates

|  | F value | *p* value |  |
| --- | --- | --- | --- |
| Fixed Effects (df) |  |  |  |
| Context (1) | 15.55 | <.001 |  |
| Chronotype (2) | 2.25 | .105 |  |
| Chronotype x Context (2) | 3.68 | .025 |  |
| Age (1) | 5.59 | .018 |  |
| Block Valence (1) | 3.70 | .055 |  |
| Block Number (1) | 6.91 | <.001 |  |
| Screen-Based Media (1) | 2.00 | .157 |  |
| Pubertal Scale (1) | 6.98 | .008 |  |
| Weekday Sleep Duration (1) | .173 | .677 |  |
| Weekend Sleep Duration (1) | 3.85 | .050 |  |
| SDQ Total Score (1) | .572 | .450 |  |
| CASQ Total Score (1) | 4.04 | .044 |  |
| Relative Social Jetlag (1) | 1.73 | .188 |  |
| Random Effects | Estimate (SE) | Z | *p* value [95% CI] |
| Participant | .993 (0.219) | 4.532 | <.001 [.644, 1.530] |
| Residual |  |  |  |
| AR1 Diagonal | 0.963 (0.021) | 45.516 | <.001 [.922, 1.005] |
| AR1 Rho | 0.032 (0.017) | 1.928 | .054 [-.001, .064] |
